# *N*-Butanoyl-*N*-(3-chloro-1,4-dioxonaph­thalen-2-yl)butanamide

**DOI:** 10.1107/S1600536813016401

**Published:** 2013-07-10

**Authors:** Ray J. Butcher, Solomon Berhe, Alan J. Anderson, Oladapo Bakare

**Affiliations:** a Department of Chemistry, Howard University, 525 College Street NW, Washington, DC 20059, USA; b Department of Natural Sciences, Bowie State University, Bowie, MD 20715, USA

## Abstract

In the title compound, C_18_H_18_ClNO_4_, the imide group with its two alkyl substituents is approximately perpendicular to the plane of the naphtho­quinone ring system [dihedral angle = 78.5 (1)°]. Further, the imide carbonyl groups are oriented in an *anti* sense. In the crystal, the substituted naphtho­quinone rings form π–π stacks in the *a-*axis direction [perpendicular centroid–centroid distance = 3.209 (2) Å and slippage = 4.401 Å].

## Related literature

For the synthesis and biological evaluation of some imido-substituted 1,4-naphtho­quinone derivatives, see; Bakare *et al.* (2003 ▶); Berhe *et al.* (2008 ▶); Brandy *et al.* (2013 ▶). For the anti-cancer and anti-trypanosomal activity of the title compound, see; Bakare *et al.* (2003 ▶); Berhe *et al.* (2008 ▶); Khraiwesh *et al.* (2012 ▶).
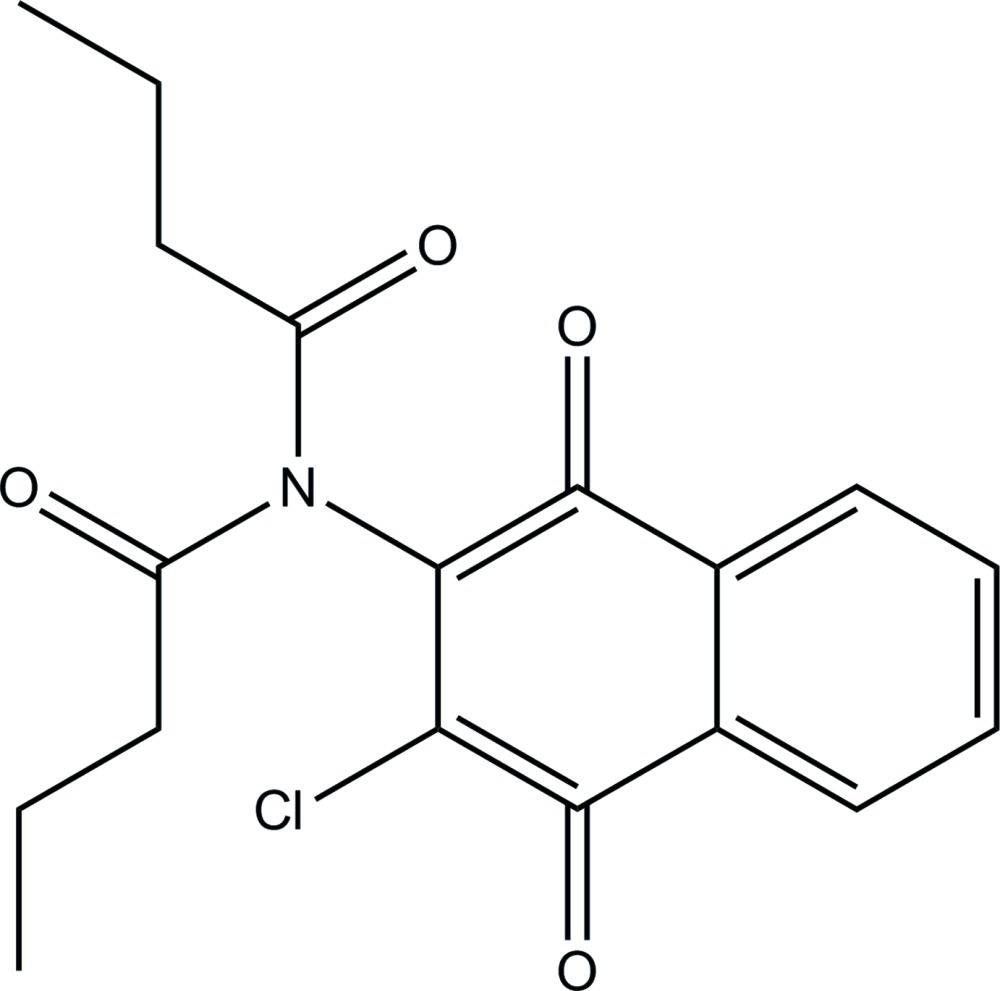


## Experimental

### 

#### Crystal data

C_18_H_18_ClNO_4_
*M* *_r_* = 347.78
Triclinic, 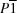

*a* = 8.1717 (10) Å
*b* = 8.3117 (10) Å
*c* = 14.6841 (15) Å
α = 93.119 (9)°
β = 98.369 (10)°
γ = 118.043 (12)°
*V* = 862.23 (17) Å^3^
*Z* = 2
Cu *K*α radiation
μ = 2.15 mm^−1^
*T* = 295 K
0.36 × 0.28 × 0.08 mm

#### Data collection

Agilent Xcalibur (Ruby, Gemini) diffractometer
Absorption correction: multi-scan (*CrysAlis PRO*; Agilent, 2012 ▶) *T*_min_ = 0.530, *T*_max_ = 1.000
5454 measured reflections
3398 independent reflections
2122 reflections with *I* > 2σ(*I*)
*R*_int_ = 0.043

#### Refinement

*R*[*F* ^2^ > 2σ(*F* ^2^)] = 0.077
*wR*(*F* ^2^) = 0.227
*S* = 1.12
3398 reflections
219 parameters
H-atom parameters constrained
Δρ_max_ = 0.39 e Å^−3^
Δρ_min_ = −0.24 e Å^−3^

### 

Data collection: *CrysAlis PRO* (Agilent, 2012 ▶); cell refinement: *CrysAlis PRO*; data reduction: *CrysAlis PRO*; program(s) used to solve structure: *SHELXS97* (Sheldrick, 2008 ▶); program(s) used to refine structure: *SHELXL97* (Sheldrick, 2008 ▶); molecular graphics: *SHELXTL* (Sheldrick, 2008 ▶); software used to prepare material for publication: *SHELXTL*.

## Supplementary Material

Crystal structure: contains datablock(s) I, global. DOI: 10.1107/S1600536813016401/hg5322sup1.cif

Structure factors: contains datablock(s) I. DOI: 10.1107/S1600536813016401/hg5322Isup2.hkl

Supplementary material file. DOI: 10.1107/S1600536813016401/hg5322Isup3.cml

Additional supplementary materials:  crystallographic information; 3D view; checkCIF report

## Figures and Tables

**Fig. 1.**
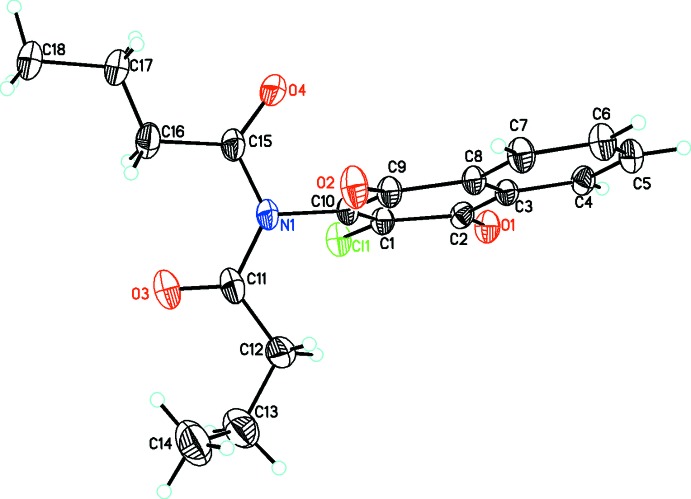
Diagram of C18H18ClNO4 showing atom labeling.

**Fig. 2.**
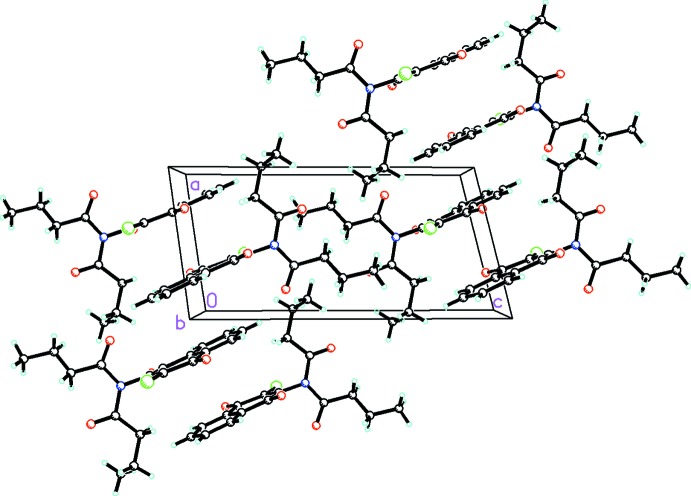
The molecular packing for C18H18ClNO4 viewed along the b axis and showing the π–π stacking in the a direction.

## supplementary crystallographic information

### Comment

We have been involved in the synthesis and biological evaluation of some imido-substituted 1,4-naphthoquinone derivatives [Bakare *et al.* (2003); Berhe *et al.* (2008); Brandy *et al.* (2013)]; and previously reported 2-chloro-3-dibutyrylamino-1,4-naphthoquinone (1) to possess inhibitory activities against certain protein kinases (Bakare *et al.* 2003). Compound 1 has subsequently been shown to possess a some desirable biological activities including anti-cancer [Bakare *et al.* (2003; Berhe *et al.* (2008)] and anti-trypanosomal activities [(Khraiwesh, *et al.*, (2012)]. We present here the crystal structure of this anticancer and antiparasitic agent.

The title compound, C_18_H_18_ClNO_4_, was synthesized as previously reported (Bakare *et al.* (2003)). The crystal structure of the title compound 1 shows that the imide group with its two alkyl substituents is almost perpendicular to the plane of the naphthoquinone ring (dihedral angle between planes of 78.5 (1)°. Further the two imide carbonyls are oriented *anti* to each other. The naphthoquinone rings form π–π stacks in the *a* direction (perpendicular *Cg*···*Cg* distance of 3.209 Å with slippage of 4.401 Å).

### Experimental

The title compound 1 was synthesized by refluxing 2-amino-3-chloro-1,4-naphthoquinone in butyryl chloride as previously reported (Bakare *et al.* (2003)). The compound was crystallized from the crude below 0°C with diethyl ether to obtain yellow crystals.

### Refinement

H atoms were placed in geometrically idealized positions and constrained to ride on their parent atoms with a C—H distances of 0.93 and 0.97 Å *U*_iso_(H) = 1.2*U*_eq_(C) and 0.96 Å for CH_3_ [*U*_iso_(H) = 1.5*U*_eq_(C)].

### Crystal data

**Table:**

C_18_H_18_ClNO_4_	*Z* = 2
*M**_r_* = 347.78	*F*(000) = 364
Triclinic, *P*1	*D*_x_ = 1.340 Mg m^−^^3^
Hall symbol: -P 1	Cu *K*α radiation, λ = 1.54178 Å
*a* = 8.1717 (10) Å	Cell parameters from 1350 reflections
*b* = 8.3117 (10) Å	θ = 3.1–75.5°
*c* = 14.6841 (15) Å	µ = 2.15 mm^−^^1^
α = 93.119 (9)°	*T* = 295 K
β = 98.369 (10)°	Plate, pale yellow
γ = 118.043 (12)°	0.36 × 0.28 × 0.08 mm
*V* = 862.23 (17) Å^3^	

### Data collection

**Table:**

Agilent Xcalibur (Ruby, Gemini) diffractometer	3398 independent reflections
Radiation source: Enhance (Cu) X-ray Source	2122 reflections with *I* > 2σ(*I*)
Graphite monochromator	*R*_int_ = 0.043
Detector resolution: 10.5081 pixels mm^-1^	θ_max_ = 75.7°, θ_min_ = 3.1°
ω scans	*h* = −8→10
Absorption correction: multi-scan (*CrysAlis PRO*; Agilent, 2012)	*k* = −10→8
*T*_min_ = 0.530, *T*_max_ = 1.000	*l* = −18→18
5454 measured reflections	

### Refinement

**Table:**

Refinement on *F*^2^	Primary atom site location: structure-invariant direct methods
Least-squares matrix: full	Secondary atom site location: difference Fourier map
*R*[*F*^2^ > 2σ(*F*^2^)] = 0.077	Hydrogen site location: inferred from neighbouring sites
*wR*(*F*^2^) = 0.227	H-atom parameters constrained
*S* = 1.12	*w* = 1/[σ^2^(*F*_o_^2^) + (0.0739*P*)^2^ + 0.4803*P*] where *P* = (*F*_o_^2^ + 2*F*_c_^2^)/3
3398 reflections	(Δ/σ)_max_ < 0.001
219 parameters	Δρ_max_ = 0.39 e Å^−^^3^
0 restraints	Δρ_min_ = −0.24 e Å^−^^3^

### Special details

**Table:**

Geometry. All e.s.d.'s (except the e.s.d. in the dihedral angle between two l.s. planes) are estimated using the full covariance matrix. The cell e.s.d.'s are taken into account individually in the estimation of e.s.d.'s in distances, angles and torsion angles; correlations between e.s.d.'s in cell parameters are only used when they are defined by crystal symmetry. An approximate (isotropic) treatment of cell e.s.d.'s is used for estimating e.s.d.'s involving l.s. planes.
Refinement. Refinement of *F*^2^ against ALL reflections. The weighted *R*-factor *wR* and goodness of fit *S* are based on *F*^2^, conventional *R*-factors *R* are based on *F*, with *F* set to zero for negative *F*^2^. The threshold expression of *F*^2^ > σ(*F*^2^) is used only for calculating *R*-factors(gt) *etc*. and is not relevant to the choice of reflections for refinement. *R*-factors based on *F*^2^ are statistically about twice as large as those based on *F*, and *R*- factors based on ALL data will be even larger.

### Fractional atomic coordinates and isotropic or equivalent isotropic displacement parameters (Å^2^)

**Table:**

	*x*	*y*	*z*	*U*_iso_*/*U*_eq_	
Cl1	0.4121 (2)	0.34538 (16)	0.17915 (8)	0.0891 (4)	
O1	0.2762 (5)	0.3535 (4)	−0.0134 (2)	0.0844 (9)	
O2	0.4177 (5)	0.9507 (4)	0.2097 (2)	0.0938 (11)	
O3	0.7195 (7)	0.7372 (8)	0.4060 (3)	0.1463 (19)	
O4	0.1712 (5)	0.5863 (5)	0.3023 (2)	0.0968 (11)	
N1	0.4736 (5)	0.6864 (5)	0.2928 (2)	0.0706 (9)	
C1	0.3740 (6)	0.5151 (5)	0.1381 (3)	0.0639 (10)	
C2	0.2990 (6)	0.4877 (5)	0.0361 (3)	0.0662 (10)	
C3	0.2519 (6)	0.6265 (5)	0.0005 (2)	0.0607 (9)	
C4	0.1688 (6)	0.6021 (6)	−0.0927 (3)	0.0722 (11)	
H4A	0.1453	0.4994	−0.1325	0.087*	
C5	0.1217 (7)	0.7286 (7)	−0.1261 (3)	0.0855 (13)	
H5A	0.0661	0.7112	−0.1883	0.103*	
C6	0.1562 (8)	0.8814 (7)	−0.0680 (3)	0.0886 (14)	
H6A	0.1230	0.9664	−0.0909	0.106*	
C7	0.2399 (7)	0.9083 (6)	0.0240 (3)	0.0785 (12)	
H7A	0.2649	1.0126	0.0628	0.094*	
C8	0.2869 (6)	0.7820 (5)	0.0590 (2)	0.0622 (9)	
C9	0.3728 (6)	0.8109 (5)	0.1584 (3)	0.0680 (10)	
C10	0.4067 (6)	0.6633 (5)	0.1942 (2)	0.0622 (10)	
C11	0.6690 (8)	0.7503 (8)	0.3266 (3)	0.0961 (16)	
C12	0.7987 (8)	0.8295 (9)	0.2605 (4)	0.1059 (18)	
H12A	0.7738	0.9213	0.2331	0.127*	
H12B	0.7675	0.7323	0.2108	0.127*	
C13	1.0076 (11)	0.9175 (12)	0.2999 (6)	0.142 (3)	
H13A	1.0310	0.8316	0.3343	0.171*	
H13B	1.0747	0.9385	0.2487	0.171*	
C14	1.0836 (12)	1.0872 (13)	0.3596 (6)	0.174 (4)	
H14A	1.2123	1.1256	0.3876	0.261*	
H14B	1.0106	1.0712	0.4073	0.261*	
H14C	1.0791	1.1792	0.3241	0.261*	
C15	0.3333 (8)	0.6480 (7)	0.3445 (3)	0.0796 (12)	
C16	0.3897 (9)	0.6902 (10)	0.4493 (3)	0.1102 (19)	
H16A	0.4304	0.6045	0.4716	0.132*	
H16B	0.4976	0.8129	0.4656	0.132*	
C17	0.2496 (10)	0.6815 (14)	0.4964 (4)	0.149 (3)	
H17A	0.1405	0.5600	0.4784	0.179*	
H17B	0.2116	0.7696	0.4751	0.179*	
C18	0.2999 (10)	0.7179 (11)	0.6009 (4)	0.131 (2)	
H18A	0.1924	0.7052	0.6254	0.197*	
H18B	0.4029	0.8406	0.6204	0.197*	
H18C	0.3366	0.6311	0.6236	0.197*	

### Atomic displacement parameters (Å^2^)

**Table:**

	*U*^11^	*U*^22^	*U*^33^	*U*^12^	*U*^13^	*U*^23^
Cl1	0.1260 (11)	0.0735 (7)	0.0734 (7)	0.0587 (7)	0.0014 (6)	0.0018 (5)
O1	0.118 (3)	0.0700 (17)	0.0620 (16)	0.0477 (18)	0.0066 (16)	−0.0132 (13)
O2	0.142 (3)	0.0725 (18)	0.0629 (17)	0.061 (2)	−0.0114 (18)	−0.0161 (14)
O3	0.135 (4)	0.212 (5)	0.079 (3)	0.083 (4)	−0.015 (2)	0.023 (3)
O4	0.100 (3)	0.121 (3)	0.0619 (18)	0.048 (2)	0.0175 (18)	−0.0028 (18)
N1	0.087 (2)	0.074 (2)	0.0479 (16)	0.0428 (19)	−0.0014 (16)	−0.0036 (14)
C1	0.075 (3)	0.058 (2)	0.057 (2)	0.0337 (19)	0.0084 (18)	0.0011 (16)
C2	0.076 (3)	0.062 (2)	0.052 (2)	0.028 (2)	0.0118 (18)	−0.0076 (16)
C3	0.065 (2)	0.062 (2)	0.0489 (18)	0.0283 (18)	0.0065 (16)	−0.0022 (15)
C4	0.083 (3)	0.077 (3)	0.048 (2)	0.035 (2)	0.0066 (19)	−0.0033 (18)
C5	0.098 (3)	0.096 (3)	0.055 (2)	0.046 (3)	0.000 (2)	0.008 (2)
C6	0.111 (4)	0.087 (3)	0.070 (3)	0.053 (3)	0.003 (3)	0.015 (2)
C7	0.101 (3)	0.069 (2)	0.063 (2)	0.045 (2)	0.001 (2)	−0.0012 (19)
C8	0.071 (2)	0.061 (2)	0.0501 (19)	0.0311 (19)	0.0041 (17)	0.0001 (15)
C9	0.084 (3)	0.061 (2)	0.052 (2)	0.034 (2)	0.0034 (18)	−0.0068 (16)
C10	0.073 (3)	0.062 (2)	0.0454 (18)	0.0316 (19)	0.0012 (16)	−0.0035 (15)
C11	0.109 (4)	0.107 (4)	0.064 (3)	0.057 (3)	−0.016 (3)	−0.006 (3)
C12	0.087 (4)	0.125 (5)	0.084 (3)	0.039 (3)	0.005 (3)	−0.009 (3)
C13	0.128 (6)	0.169 (7)	0.131 (6)	0.084 (6)	−0.009 (5)	0.007 (5)
C14	0.145 (7)	0.175 (8)	0.126 (6)	0.035 (6)	−0.032 (5)	0.002 (6)
C15	0.101 (4)	0.087 (3)	0.054 (2)	0.052 (3)	0.003 (2)	−0.003 (2)
C16	0.131 (5)	0.149 (5)	0.056 (3)	0.075 (4)	0.011 (3)	0.001 (3)
C17	0.131 (5)	0.253 (9)	0.055 (3)	0.090 (6)	0.014 (3)	−0.003 (4)
C18	0.140 (6)	0.185 (7)	0.057 (3)	0.072 (5)	0.015 (3)	−0.002 (4)

### Geometric parameters (Å, º)

**Table:**

Cl1—C1	1.703 (4)	C9—C10	1.484 (5)
O1—C2	1.218 (4)	C11—C12	1.487 (8)
O2—C9	1.215 (4)	C12—C13	1.508 (8)
O3—C11	1.207 (6)	C12—H12A	0.9700
O4—C15	1.221 (6)	C12—H12B	0.9700
N1—C15	1.389 (6)	C13—C14	1.424 (10)
N1—C11	1.422 (6)	C13—H13A	0.9700
N1—C10	1.440 (4)	C13—H13B	0.9700
C1—C10	1.335 (5)	C14—H14A	0.9600
C1—C2	1.496 (5)	C14—H14B	0.9600
C2—C3	1.475 (6)	C14—H14C	0.9600
C3—C4	1.395 (5)	C15—C16	1.512 (6)
C3—C8	1.397 (5)	C16—C17	1.397 (8)
C4—C5	1.369 (6)	C16—H16A	0.9700
C4—H4A	0.9300	C16—H16B	0.9700
C5—C6	1.377 (7)	C17—C18	1.505 (7)
C5—H5A	0.9300	C17—H17A	0.9700
C6—C7	1.377 (6)	C17—H17B	0.9700
C6—H6A	0.9300	C18—H18A	0.9600
C7—C8	1.376 (6)	C18—H18B	0.9600
C7—H7A	0.9300	C18—H18C	0.9600
C8—C9	1.479 (5)		
			
C15—N1—C11	127.5 (4)	C13—C12—H12A	108.1
C15—N1—C10	113.5 (4)	C11—C12—H12B	108.1
C11—N1—C10	119.0 (4)	C13—C12—H12B	108.1
C10—C1—C2	121.8 (4)	H12A—C12—H12B	107.3
C10—C1—Cl1	121.9 (3)	C14—C13—C12	114.9 (7)
C2—C1—Cl1	116.3 (3)	C14—C13—H13A	108.5
O1—C2—C3	122.9 (4)	C12—C13—H13A	108.5
O1—C2—C1	120.0 (4)	C14—C13—H13B	108.5
C3—C2—C1	117.0 (3)	C12—C13—H13B	108.5
C4—C3—C8	119.0 (4)	H13A—C13—H13B	107.5
C4—C3—C2	119.9 (3)	C13—C14—H14A	109.5
C8—C3—C2	121.1 (3)	C13—C14—H14B	109.5
C5—C4—C3	120.4 (4)	H14A—C14—H14B	109.5
C5—C4—H4A	119.8	C13—C14—H14C	109.5
C3—C4—H4A	119.8	H14A—C14—H14C	109.5
C4—C5—C6	120.4 (4)	H14B—C14—H14C	109.5
C4—C5—H5A	119.8	O4—C15—N1	117.7 (4)
C6—C5—H5A	119.8	O4—C15—C16	123.7 (5)
C5—C6—C7	119.9 (4)	N1—C15—C16	118.6 (5)
C5—C6—H6A	120.0	C17—C16—C15	115.8 (5)
C7—C6—H6A	120.0	C17—C16—H16A	108.3
C8—C7—C6	120.6 (4)	C15—C16—H16A	108.3
C8—C7—H7A	119.7	C17—C16—H16B	108.3
C6—C7—H7A	119.7	C15—C16—H16B	108.3
C7—C8—C3	119.8 (4)	H16A—C16—H16B	107.4
C7—C8—C9	119.9 (3)	C16—C17—C18	117.0 (6)
C3—C8—C9	120.3 (4)	C16—C17—H17A	108.1
O2—C9—C8	122.0 (4)	C18—C17—H17A	108.1
O2—C9—C10	120.3 (4)	C16—C17—H17B	108.1
C8—C9—C10	117.6 (3)	C18—C17—H17B	108.1
C1—C10—N1	121.5 (4)	H17A—C17—H17B	107.3
C1—C10—C9	121.9 (3)	C17—C18—H18A	109.5
N1—C10—C9	116.6 (3)	C17—C18—H18B	109.5
O3—C11—N1	118.8 (6)	H18A—C18—H18B	109.5
O3—C11—C12	124.2 (6)	C17—C18—H18C	109.5
N1—C11—C12	117.0 (4)	H18A—C18—H18C	109.5
C11—C12—C13	116.7 (5)	H18B—C18—H18C	109.5
C11—C12—H12A	108.1		
			
C10—C1—C2—O1	177.8 (4)	C2—C1—C10—C9	−0.7 (6)
Cl1—C1—C2—O1	−3.1 (6)	Cl1—C1—C10—C9	−179.8 (3)
C10—C1—C2—C3	−3.5 (6)	C15—N1—C10—C1	−102.5 (5)
Cl1—C1—C2—C3	175.6 (3)	C11—N1—C10—C1	79.8 (5)
O1—C2—C3—C4	3.2 (6)	C15—N1—C10—C9	76.4 (5)
C1—C2—C3—C4	−175.4 (4)	C11—N1—C10—C9	−101.2 (5)
O1—C2—C3—C8	−177.6 (4)	O2—C9—C10—C1	−174.2 (4)
C1—C2—C3—C8	3.7 (6)	C8—C9—C10—C1	4.7 (6)
C8—C3—C4—C5	−0.3 (7)	O2—C9—C10—N1	6.8 (6)
C2—C3—C4—C5	178.9 (4)	C8—C9—C10—N1	−174.3 (4)
C3—C4—C5—C6	0.1 (8)	C15—N1—C11—O3	16.4 (8)
C4—C5—C6—C7	0.5 (8)	C10—N1—C11—O3	−166.3 (5)
C5—C6—C7—C8	−1.1 (8)	C15—N1—C11—C12	−163.7 (5)
C6—C7—C8—C3	0.9 (7)	C10—N1—C11—C12	13.6 (7)
C6—C7—C8—C9	−178.6 (5)	O3—C11—C12—C13	−7.2 (10)
C4—C3—C8—C7	−0.3 (6)	N1—C11—C12—C13	172.9 (5)
C2—C3—C8—C7	−179.4 (4)	C11—C12—C13—C14	−70.9 (9)
C4—C3—C8—C9	179.3 (4)	C11—N1—C15—O4	−176.9 (4)
C2—C3—C8—C9	0.1 (6)	C10—N1—C15—O4	5.7 (6)
C7—C8—C9—O2	−5.9 (7)	C11—N1—C15—C16	5.1 (7)
C3—C8—C9—O2	174.5 (4)	C10—N1—C15—C16	−172.3 (4)
C7—C8—C9—C10	175.3 (4)	O4—C15—C16—C17	−9.5 (10)
C3—C8—C9—C10	−4.3 (6)	N1—C15—C16—C17	168.4 (6)
C2—C1—C10—N1	178.2 (4)	C15—C16—C17—C18	178.4 (7)
Cl1—C1—C10—N1	−0.9 (6)		
